# Supplementary material for: Dietary Liberalization in Tetrahydrobiopterin-Treated PKU Patients: Does It Improve Outcomes?
Source: Nutrients. 2022 Sep 19;14(18):3874. doi: 10.3390/nu14183874 (PMC9504284; doi:10.3390/nu14183874)
Supplement: Supplementary file 1 [file nutrients-14-03874-s001.zip › Supplementary file S2 - Search details.pdf]

Supplementary file S2 - Search strategy

Databases
Three databases were searched:
1. Pubmed
2. Cochrane
3. Embase (Ovid)

A combination was used of:
- Mesh terms (Medical Subject Headings) from Pubmed and Cochrane or Emtree terms from Embase
- Text-word search in title, abstract and author keywords.

Entry terms
1. "phenylketonurias"[Mesh]
2. "phenylalanine hydroxylase/deficiency"[Mesh]
3. hyperphenylalaninaemia OR hyperphenylalaninemia
4. phenylalanine hydroxylase deficiency OR PAH deficiency
5. phenylketonuri*
6. pku
7. 1 or 2 or 3 or 4 or 5 or 6
8. "sapropterin" [Supplementary Concept]
9. tetrahydrobiopterin
10. bh4 or bh 4
11. sapropterin
12. kuvan
13. 8 or 9 or 10 or 11 or 12
21. 7 and 13

For Embase the following publications types were excluded: books or chapter or conference abstract or editorial or note or tombstone

Syntax of Mesh/Emtree terms per database

Entry nr	Pubmed	Cochrane	Embase (OVID)
1	"phenylketonurias"[Mesh]	MeSH descriptor: [phenylketonurias] explode all trees	exp phenylketonuria/<br>exp hyperphenylalaninemia/
2	"phenylalanine hydroxylase/deficiency"[Mesh]	MeSH descriptor: Phenylalanine Hydroxylase] explode all trees and with qualifier(s): [deficiency – DF]	exp phenylalanine 4 monooxygenase/ and deficiency.ti.ab.kw
8	"sapropterin" [Supplementary Concept]	/	exp tetrahydrobiopterin/<br>exp sapropterin/

Syntax of title, abstract and author keyword per database

Entry nr	Pubmed	Cochrane	Embase (OVID)
3-6, 14-23	[tiab]	:ti,ab,kw	.ti,ab,kw.
