# Supplementary material for: Dietary Liberalization in Tetrahydrobiopterin-Treated PKU Patients: Does It Improve Outcomes?
Source: Nutrients. 2022 Sep 19;14(18):3874. doi: 10.3390/nu14183874 (PMC9504284; doi:10.3390/nu14183874)
Supplement: Supplementary file 1 [file nutrients-14-03874-s001.zip › Supplementary file S3 - Meta-analyses.pdf]

Supplementary file S3 - Overview of meta-analyses

Parameter		Primary meta-analyses (longest follow-up time per study)				Secondary meta-analyses: Short-term effects (≤ 6 months of follow up time)			Secondary meta-analyses: Long-term effects (≥ 5 years of follow up time)		
		Overall	Stratified by cohort age			Overall	Stratified by cohort age		Overall	Stratified by cohort age	
			< 12	12-18	> 18		< 12	12-18		< 12	12-18
**Weight**	Within-subject	p = 0.61	p = 0.08 (2)	p = 0.39 (1)		*p = 0.58*	*p = 0.58 (2)*		p = 0.50	p = 0.97 (1)	p = 0.39 (1)
	Between-subject	p = 0.95	p = 0.33 (2)	p = 0.27 (1)		***p = 0.01***	***p = 0.01 (2)***		p = 0.32	p = 0.83 (1)	p = 0.27 (1)
**BMI**	Within-subject	p = 0.82	p = 0.97 (2)	p = 0.71 (1)		*p = 0.69*	*p = 0.69 (3)*		p = 0.85	p = 0.83 (1)	p = 0.71 (1)
	Between-subject	***p = 0.02***	***p = 0.02 (2)***	p = 0.57 (1)		*p = 0.38*	*p = 0.38 (2)*		p = 0.47	p = 0.65 (1)	p = 0.57 (1)
**Height**	Within-subject	p = 0.80	p = 0.91 (3)	p = 0.72 (1)		*p = 0.84*	*p = 0.84 (3)*		p = 0.58	p = 0.66 (1)	p = 0.72 (1)
	Between-subject	p = 0.43	p = 0.43 (2)	p = 0.86 (1)		p = 0.57	p = 0.57 (2)		p = 0.85	p = 0.92 (1)	p = 0.86 (1)
**Growth velocity**	Within-subject	p = 0.20	p = 0.42 (2)	p = 0.25 (1)		p = 0.49	p = 0.49 (2)		p = 0.06	p = 0.13 (1)	p = 0.25 (1)
	Between-subject	p = 0.73	p = 0.96 (2)	p = 0.40 (1)		p = 0.77	p = 0.77 (2)		p = 0.64	p = 0.93 (1)	p = 0.40 (1)
**Albumin**	Within-subject	p = 0.40	p = 0.58 (2)	p = 0.65 (1)							
**Cholesterol**	Within-subject	***p = 0.01***	p = 0.52 (1)	***p = 0.01 (1)***							
**Haemoglobin**	Within-subject	p = 0.11	p = 0.27 (1)	p = 0.22 (1)							
**HR-QoL (patient-report)**	Within-subject	p = 0.86	p = 0.97 (1)	p = 0.83 (2)		p = 0.83	p = 0.97 (1)	p = 0.82 (1)			
	Between-subject	p = 0.92	p = 0.68 (1)	p = 0.92 (2)		p = 0.95	p = 0.68 (1)	p = 0.75 (1)			
**HR-QoL (proxy-report)**	Within-subject	p = 0.14	p = 0.78 (2)	p = 0.09 (1)		p = 0.15					
	Between-subject	p = 0.22	p = 0.38 (2)	p = 0.40 (1)		p = 0.32					

Table S3.1. Overview of all meta-analyses performed in this systematic review. Results are given as p-values (number of cohorts). Stratifications were made according to mean cohort age in years. BMI, body mass index. HR-QoL, health-related quality of life. IQ, intelligent quotient.
