# Supplementary material for: Dietary Liberalization in Tetrahydrobiopterin-Treated PKU Patients: Does It Improve Outcomes?
Source: Nutrients. 2022 Sep 19;14(18):3874. doi: 10.3390/nu14183874 (PMC9504284; doi:10.3390/nu14183874)
Supplement: Supplementary file 1 [file nutrients-14-03874-s001.zip › Supplementary file S4 - Forest plot of primary meta-analyses.pdf]

Supplementary file S4 – Forest plots of primary meta-analyses

Study or Subgroup	Before start BH4 Mean	SD	Total	After start BH4 Mean	SD	Total	Weight	Std. Mean Difference IV, Random, 95% CI
Aldámiz-Echevarría 2013 (1)	-0.16	1.03	36	-0.75	1.04	36	41.4%	0.56 [0.09, 1.04]
Aldámiz-Echevarría 2013 (2)	-0.08	1.08	10	-0.1	1.01	10	24.0%	0.02 [-0.86, 0.89]
Evers 2018	0.86	1.19	21	1.21	1.4	21	34.6%	-0.26 [-0.87, 0.34]
**Total (95% CI)**			**67**			**67**	**100.0%**	**0.15 [-0.41, 0.70]**

Heterogeneity: Tau² = 0.14; Chi² = 4.69, df = 2 (P = 0.10); I² = 57%
Test for overall effect: Z = 0.51 (P = 0.61)

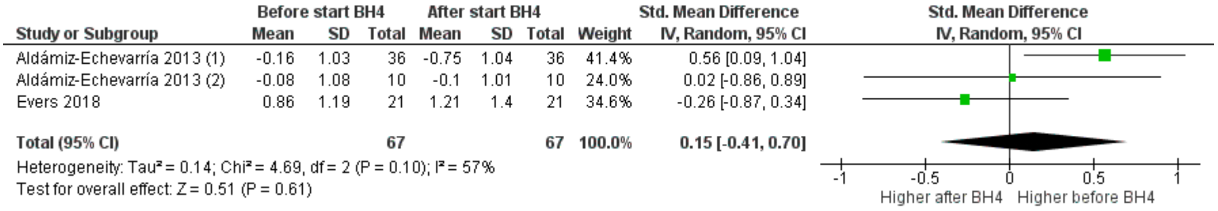

Figure S4.1. Forest plot of the primary within-subject meta-analysis for weight.

Study or Subgroup	Control group Mean	SD	Total	BH4-treated group Mean	SD	Total	Weight	Std. Mean Difference IV, Random, 95% CI
Aldámiz-Echevarría 2013 (1)	-0.52	0.85	72	-0.75	1.04	36	51.1%	0.25 [-0.15, 0.65]
Aldámiz-Echevarría 2013 (2)	-0.18	0.88	20	-0.1	1.01	10	20.1%	-0.08 [-0.84, 0.68]
Evers 2018	0.67	1.68	21	1.21	1.4	21	28.7%	-0.34 [-0.95, 0.27]
**Total (95% CI)**			**113**			**67**	**100.0%**	**0.01 [-0.36, 0.38]**

Heterogeneity: Tau² = 0.03; Chi² = 2.66, df = 2 (P = 0.27); I² = 25%
Test for overall effect: Z = 0.06 (P = 0.95)

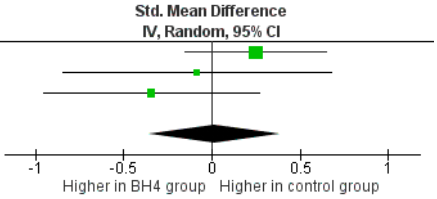

Figure S4.2. Forest plot of the primary between-subject meta-analysis for weight.

Study or Subgroup	Before starting BH4 Mean	SD	Total	After starting BH4 Mean	SD	Total	Weight	Std. Mean Difference IV, Random, 95% CI
Singh 2010	0.68	0.57	6	0.62	0.42	6	8.2%	0.11 [-1.02, 1.24]
Aldámiz-Echevarría 2013 (1)	0.31	1.03	36	0.37	1.09	36	49.4%	-0.06 [-0.52, 0.41]
Aldámiz-Echevarría 2013 (2)	0.23	1.1	10	0.12	1.03	10	13.7%	0.10 [-0.78, 0.98]
Evers 2018	0.81	1.07	21	0.94	1.13	21	28.7%	-0.12 [-0.72, 0.49]
**Total (95% CI)**			**73**			**73**	**100.0%**	**-0.04 [-0.36, 0.29]**

Heterogeneity: Tau² = 0.00; Chi² = 0.23, df = 3 (P = 0.97); I² = 0%
Test for overall effect: Z = 0.23 (P = 0.82)

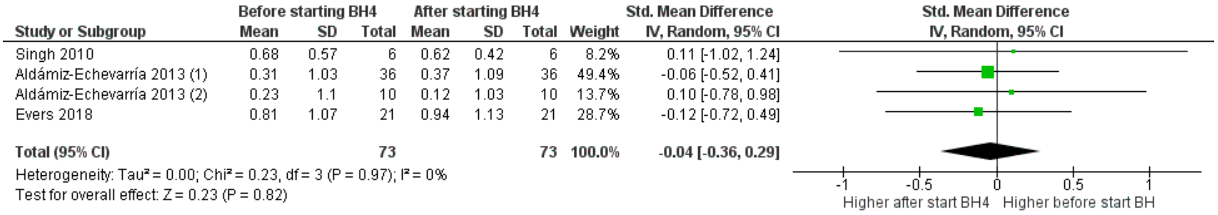

Figure S4.3. Forest plot of the primary within-subject meta-analysis for BMI.

Study or Subgroup	Control group Mean	SD	Total	BH4-treated group Mean	SD	Total	Weight	Std. Mean Difference IV, Random, 95% CI
Aldámiz-Echevarría 2013 (1)	-0.12	0.89	72	0.37	1.09	36	57.7%	-0.51 [-0.91, -0.10]
Aldámiz-Echevarría 2013 (2)	-0.04	0.8	20	0.12	1.03	10	16.4%	-0.18 [-0.94, 0.58]
Evers 2018	0.71	1.43	21	0.94	1.13	21	25.9%	-0.18 [-0.78, 0.43]
**Total (95% CI)**			**113**			**67**	**100.0%**	**-0.37 [-0.67, -0.06]**

Heterogeneity: Tau² = 0.00; Chi² = 1.08, df = 2 (P = 0.58); I² = 0%
Test for overall effect: Z = 2.33 (P = 0.02)

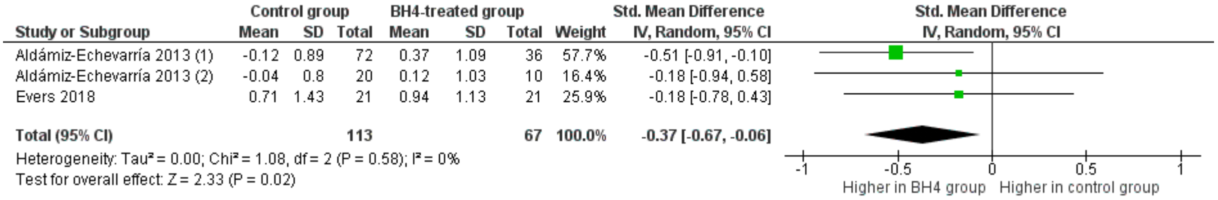

Figure S4.4. Forest plot of the primary between-subject meta-analysis for BMI.

Study or Subgroup	Before start BH4 Mean	SD	Total	After start BH4 Mean	SD	Total	Weight	Std. Mean Difference IV, Random, 95% CI
Singh 2010	0.25	0.99	6	0.53	1.16	6	9.3%	-0.24 [-1.38, 0.90]
Aldámiz-Echevarría 2013 (1)	-0.71	1.25	36	-0.73	0.96	36	56.1%	0.02 [-0.44, 0.48]
Aldámiz-Echevarría 2013 (2)	-0.29	1.31	10	-0.52	0.9	10	15.5%	0.20 [-0.68, 1.08]
Evers 2018	0.2	1.03	16	0.01	1.63	10	19.1%	0.14 [-0.65, 0.93]
**Total (95% CI)**			**68**			**62**	**100.0%**	**0.05 [-0.30, 0.39]**

Heterogeneity: Tau² = 0.00; Chi² = 0.43, df = 3 (P = 0.93); I² = 0%
Test for overall effect: Z = 0.26 (P = 0.80)

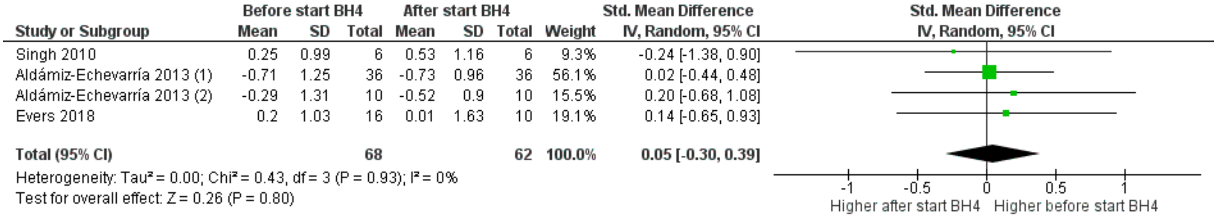

Figure S4.5. Forest plot of the primary within-subject meta-analysis for height.

Study or Subgroup	Control group Mean	SD	Total	BH4-treated group Mean	SD	Total	Weight	Std. Mean Difference IV, Random, 95% CI
Aldámiz-Echevarría 2013 (1)	-0.9	1	72	-0.73	0.96	36	67.7%	-0.17 [-0.57, 0.23]
Aldámiz-Echevarría 2013 (2)	-0.56	1.09	20	-0.52	0.9	10	18.9%	-0.04 [-0.80, 0.72]
Evers 2018	-0.11	1.15	9	0.01	1.63	10	13.4%	-0.08 [-0.98, 0.82]
**Total (95% CI)**			**101**			**56**	**100.0%**	**-0.13 [-0.46, 0.20]**

Heterogeneity: Tau² = 0.00; Chi² = 0.11, df = 2 (P = 0.95); I² = 0%
Test for overall effect: Z = 0.79 (P = 0.43)

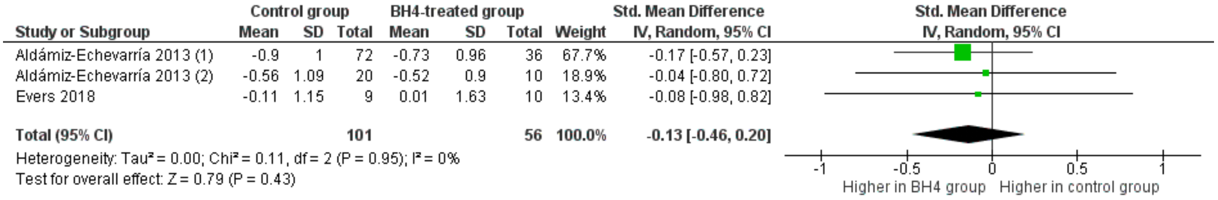

Figure S4.6. Forest plot of the primary between-subject meta-analysis for height.

Study or Subgroup	Before start BH4 Mean	SD	Total	After start BH4 Mean	SD	Total	Weight	Std. Mean Difference IV, Random, 95% CI
Aldámiz-Echevarría 2013 (1)	0.15	2.18	36	0.05	2.71	36	62.2%	0.04 [-0.42, 0.50]
Aldámiz-Echevarría 2013 (2)	1.62	1.62	10	0.41	1.67	10	16.6%	0.70 [-0.21, 1.61]
Evers 2018	0.08	1.31	16	-0.62	1.63	10	21.2%	0.47 [-0.33, 1.27]
**Total (95% CI)**			**62**			**56**	**100.0%**	**0.24 [-0.13, 0.61]**

Heterogeneity: Tau^2 = 0.00; Chi^2 = 2.04, df = 2 (P = 0.36); I^2 = 2%
Test for overall effect: Z = 1.27 (P = 0.20)

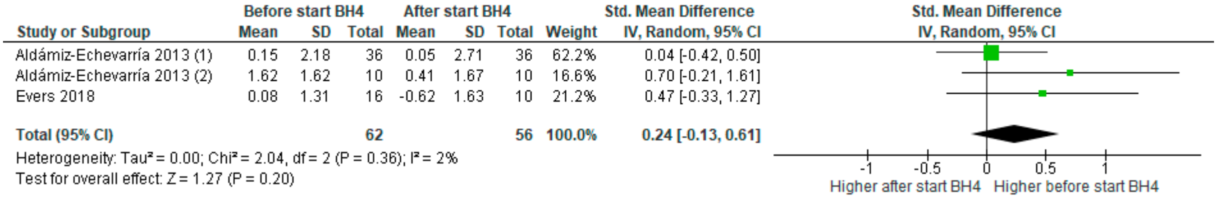

Figure S4.7. Forest plot of the primary within-subject meta-analysis for growth velocity.

Study or Subgroup	Control group Mean	SD	Total	BH4-treated group Mean	SD	Total	Weight	Std. Mean Difference IV, Random, 95% CI
Aldámiz-Echevarría 2013 (1)	0.1	2.26	72	0.05	2.71	36	68.0%	0.02 [-0.38, 0.42]
Aldámiz-Echevarría 2013 (2)	0.34	2.1	20	0.41	1.67	10	18.9%	-0.03 [-0.79, 0.72]
Evers 2018	0.27	2.69	9	-0.62	1.63	10	13.1%	0.39 [-0.52, 1.30]
**Total (95% CI)**			**101**			**56**	**100.0%**	**0.06 [-0.27, 0.39]**

Heterogeneity: Tau^2 = 0.00; Chi^2 = 0.59, df = 2 (P = 0.74); I^2 = 0%
Test for overall effect: Z = 0.35 (P = 0.73)

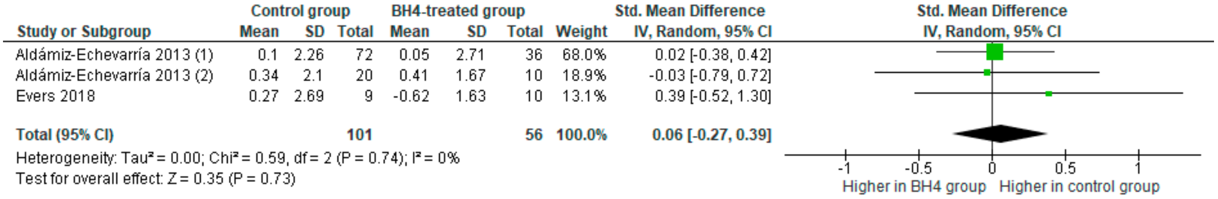

Figure S4.8. Forest plot of the primary between-subject meta-analysis for growth velocity.

Study or Subgroup	Before BH4 treatment Mean	SD	Total	After BH4 treatment Mean	SD	Total	Weight	Std. Mean Difference IV, Random, 95% CI	Year
Lambruschini 2005	46	5	11	45	3	11	28.9%	0.23 [-0.61, 1.07]	2005
Singh 2010	4.7	0.1	6	4.6	0.4	6	15.6%	0.32 [-0.83, 1.46]	2010
Evers 2018	47.53	2.92	21	47.11	3.01	21	55.5%	0.14 [-0.47, 0.74]	2018
**Total (95% CI)**			**38**			**38**	**100.0%**	**0.19 [-0.26, 0.65]**	

Heterogeneity: Tau^2 = 0.00; Chi^2 = 0.08, df = 2 (P = 0.96); I^2 = 0%
Test for overall effect: Z = 0.84 (P = 0.40)

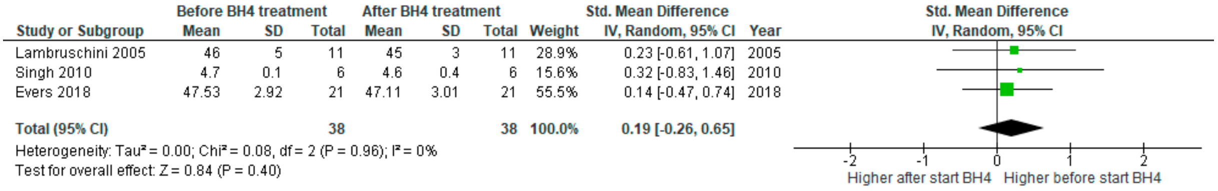

Figure S4.9. Forest plot of the primary within-subject meta-analysis for blood albumin concentrations.

Study or Subgroup	Before BH4 treatment Mean	SD	Total	After BH4 treatment Mean	SD	Total	Weight	Std. Mean Difference IV, Random, 95% CI	Year
Singh 2010	131	9.9	6	137	18.3	6	23.3%	-0.38 [-1.52, 0.77]	2010
Evers 2018	3.42	0.46	21	3.8	0.47	21	76.7%	-0.80 [-1.43, -0.17]	2018
**Total (95% CI)**			**27**			**27**	**100.0%**	**-0.70 [-1.26, -0.15]**	

Heterogeneity: Tau^2 = 0.00; Chi^2 = 0.41, df = 1 (P = 0.52); I^2 = 0%
Test for overall effect: Z = 2.49 (P = 0.01)

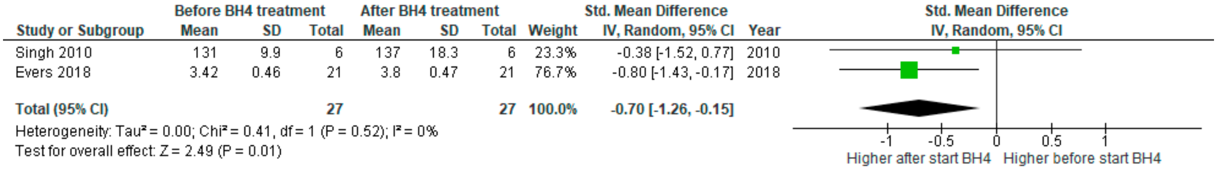

Figure S4.10. Forest plot of the primary within-subject meta-analysis for blood cholesterol concentrations.

Study or Subgroup	Before BH4 treatment Mean	SD	Total	After BH4 treatment Mean	SD	Total	Weight	Std. Mean Difference IV, Random, 95% CI	Year
Singh 2010	13.7	0.7	6	14.4	1.2	6	21.2%	-0.66 [-1.83, 0.52]	2010
Evers 2018	134.7	15.5	21	140.11	12.33	21	78.8%	-0.38 [-0.99, 0.23]	2018
**Total (95% CI)**			**27**			**27**	**100.0%**	**-0.44 [-0.98, 0.10]**	

Heterogeneity: Tau^2 = 0.00; Chi^2 = 0.17, df = 1 (P = 0.68); I^2 = 0%
Test for overall effect: Z = 1.58 (P = 0.11)

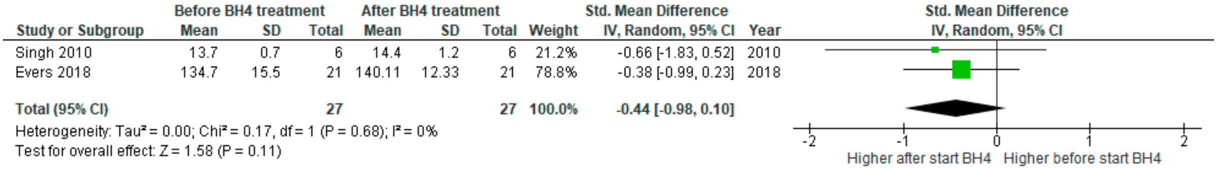

Figure S4.11. Forest plot of the primary within-subject meta-analysis for blood haemoglobin concentrations.

Study or Subgroup	Before starting BH4 Mean	SD	Total	After starting BH4 Mean	SD	Total	Weight	Std. Mean Difference IV, Random, 95% CI	Year
Ziesch 2012	79.6	9.5	8	79.8	9.5	8	24.6%	-0.02 [-1.00, 0.96]	2012
Demirdas 2013 (8-17 years old)	91.3	6.4	5	87.2	4.9	5	14.1%	0.65 [-0.64, 1.94]	2013
Feldmann 2017	74.5	11.2	20	75.3	10.3	20	61.3%	-0.07 [-0.69, 0.55]	2017
**Total (95% CI)**			**33**			**33**	**100.0%**	**0.04 [-0.44, 0.53]**	

Heterogeneity: Tau^2 = 0.00; Chi^2 = 1.00, df = 2 (P = 0.61); I^2 = 0%
Test for overall effect: Z = 0.17 (P = 0.86)

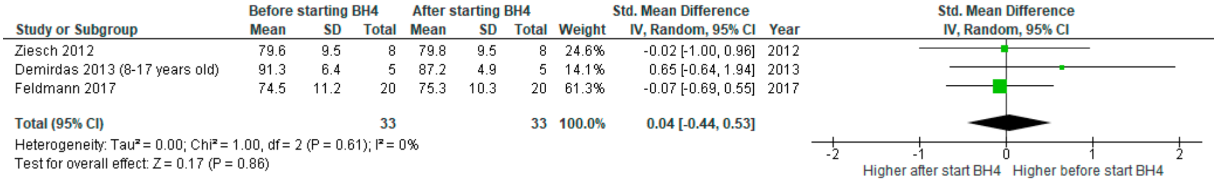

Figure S4.12. Forest plot of the primary within-subject meta-analysis for generic health-related quality of life – patient report.

Study or Subgroup	Control group Mean	SD	Total	BH4-treated group Mean	SD	Total	Weight	Std. Mean Difference IV, Random, 95% CI	Year
Ziesch 2012	77.4	10.4	6	79.8	9.5	8	20.6%	-0.23 [-1.29, 0.84]	2012
Demirdas 2013 (8-17 years old)	85	14.2	14	87.2	4.9	5	22.2%	-0.17 [-1.19, 0.86]	2013
Feldmann 2017	76.6	13.8	18	75.3	10.3	20	57.2%	0.11 [-0.53, 0.74]	2017
**Total (95% CI)**			**38**			**33**	**100.0%**	**-0.02 [-0.51, 0.46]**	

Heterogeneity: Tau² = 0.00; Chi² = 0.37, df = 2 (P = 0.83); I² = 0%
Test for overall effect: Z = 0.09 (P = 0.92)

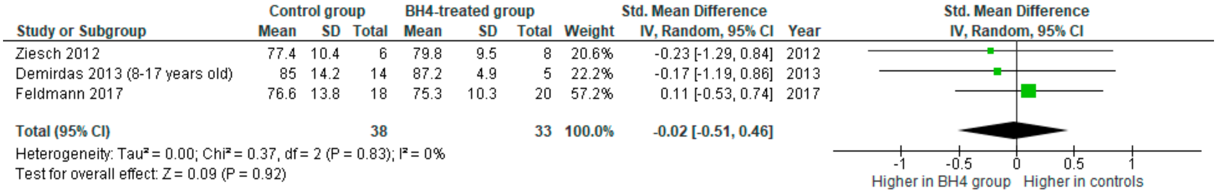

Figure S4.13. Forest plot of the primary between-subject meta-analysis for generic health-related quality of life – patient report.

Study or Subgroup	Before starting BH4 Mean	SD	Total	After starting BH4 Mean	SD	Total	Weight	Std. Mean Difference IV, Random, 95% CI	Year
Ziesch 2012	79.5	7.6	8	79.6	11	8	24.9%	-0.01 [-0.99, 0.97]	2012
Demirdas 2013 (4-17 years old)	84.2	13.4	6	87.1	8.4	6	18.5%	-0.24 [-1.38, 0.90]	2013
Feldmann 2017	70.4	14.2	19	77.3	9.1	19	56.6%	-0.57 [-1.22, 0.08]	2017
**Total (95% CI)**			**33**			**33**	**100.0%**	**-0.37 [-0.86, 0.12]**	

Heterogeneity: Tau² = 0.00; Chi² = 0.92, df = 2 (P = 0.63); I² = 0%
Test for overall effect: Z = 1.47 (P = 0.14)

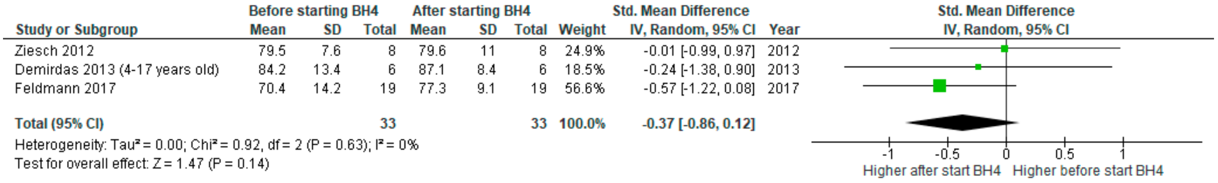

Figure S4.14. Forest plot of the primary within-subject meta-analysis for generic health-related quality of life – proxy report.

Study or Subgroup	Control group Mean	SD	Total	BH4-treated group Mean	SD	Total	Weight	Std. Mean Difference IV, Random, 95% CI	Year
Ziesch 2012	77	4.4	6	79.6	11	8	19.5%	-0.27 [-1.34, 0.79]	2012
Demirdas 2013 (4-17 years old)	81.7	16.6	20	87.1	8.4	6	26.3%	-0.34 [-1.26, 0.58]	2013
Feldmann 2017	74.3	11.9	19	77.3	9.1	19	54.2%	-0.28 [-0.92, 0.36]	2017
**Total (95% CI)**			**45**			**33**	**100.0%**	**-0.29 [-0.76, 0.18]**	

Heterogeneity: Tau² = 0.00; Chi² = 0.01, df = 2 (P = 0.99); I² = 0%
Test for overall effect: Z = 1.22 (P = 0.22)

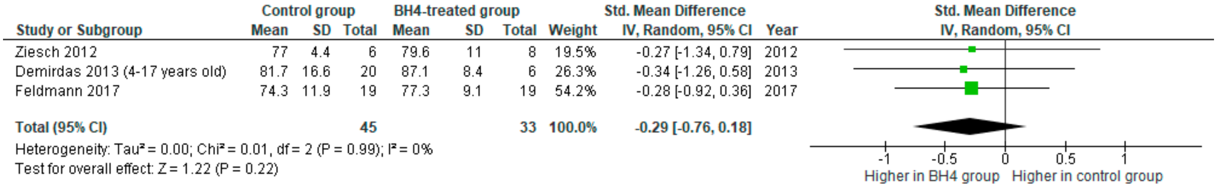

Figure S4.15. Forest plot of the primary between-subject meta-analysis for generic health-related quality of life – proxy report.
