# Supplementary material for: Dietary Liberalization in Tetrahydrobiopterin-Treated PKU Patients: Does It Improve Outcomes?
Source: Nutrients. 2022 Sep 19;14(18):3874. doi: 10.3390/nu14183874 (PMC9504284; doi:10.3390/nu14183874)
Supplement: Supplementary file 1 [file nutrients-14-03874-s001.zip › Supplementary file S5 - Excluded studies - origineel.pdf]

Supplementary file S5 – Excluded studies

Reasons for exclusion:
1. No full text available
2. Not written in English
3. Wrong publication type (conference abstract, review, expert opinion, case reports)
4. No outcome of interest (Phe levels, Tyr levels, dietary intake and other surrogate markers)
5. Wrong study design (cross-sectional study, cross-sectional data for outcome of interest)
6. No quantitative data / insufficient data
7. No dietary liberalization

Study / Year	Reason for exclusion
Aldámiz-Echevarría 2015 [1]	Overlapping study data
Anjema 2013 [2]	No outcome of interest
Anjema 2016 [3]	No outcome of interest
Baldellou Vazquez 2006 [4]	No full text available, Not written in English
Bélanger-Quintana 2005 [5]	No outcome of interest
Boveda 2007 [6]	No outcome of interest
Bushueva 2014 [7]	No full text available, Not written in English
Burlina 2009 [8]	No outcome of interest
Burton 2007 [9]	No outcome of interest
Burton 2010 [10]	No outcome of interest
Burton 2011 [11]	No outcome of interest
Burton 2015 [12]	No dietary liberalization
Campistol 2012 [13]	No full text available, Not written in English
Cazzorla 2014 [14]	Wrong study design (cross-sectional)
Christ 2013 [15]	No dietary liberalization
Clocksin 2021 [16]	No dietary liberalization
Cohen-Pfeffer 2015 [17]	Wrong publication type (SSIEM conference abstract)
Couce 2012 [18]	Wrong study design (only current IQ)
Doggrell 2008 [19]	Wrong publication type (expert opinion)
Douglas 2013 [20]	No outcome of interest
Evans 2017 [21]	Wrong study design (cross-sectional)
Feillet 2014 [22]	Wrong publication type (MPKU cases)
Ficicioglu 2013 [23]	No dietary liberalization
Gassio 2010 [24]	Wrong study design (cross-sectional)
Grange 2014 [25]	Wrong publication type (MPKU cases)
Hennermann 2005 [26]	No quantitative data / insufficient data
Hennermann 2012 [27]	No outcome of interest
Huijbregts 2018 [28]	Wrong study design (cross-sectional)
Humphrey 2011 [29]	No outcome of interest
Kagnici 2014 [30]	Wrong publication type (SSIEM conference abstract)
Keil 2013 [31]	No quantitative data / insufficient data
Kör 2017 [32]	No outcome of interest

Lee 2008 [33]	No outcome of interest
Leuret 2012 [34]	No outcome of interest
Levy 2007 [35]	No outcome of interest
Lindegren 2013 [36]	No outcome of interest (systematic review)
Longo 2015 [37]	Overlapping study data
Longo 2015 [38]	No outcome of interest
Moseley 2015 [39]	No dietary liberalization
Muntau 2002 [40]	No outcome of interest
Muntau 2014 [41]	Wrong publication type (SSIEM conference abstract)
Muntau 2016 [42]	Wrong publication type (SSIEM conference abstract)
Muntau 2017 [43]	Overlapping study data
Ormazabal 2005 [44]	No outcome of interest
Prasad 2013 [45]	Wrong publication type (ICIEM conference abstract)
Qu 2019 [46]	No outcome of interest (meta-analysis)
Rutsch 2018 [47]	Wrong publication type (SSIEM conference abstract)
Shintaku 2004 [48]	No outcome of interest
Shintaku 2005 [49]	No full text available, Not written in English
Shintaku 2014 [50]	No outcome of interest
Singh 2011 [51]	No outcome of interest
Somaraju 2009 [52]	No outcome of interest (systematic review)
Somaraju 2010 [53]	No outcome of interest (systematic review)
Somaraju 2012 [54]	No outcome of interest (systematic review)
Somaraju 2015 [55]	No outcome of interest (systematic review)
Sousa Barbossa 2018 [56]	No outcome of interest
Stockler-Ipsiroglu 2015 [57]	No outcome of interest
Tamura 2022 [58]	No quantitative data / insufficient data
Thiele 2013 [59]	No outcome of interest
Thiele 2015 [60]	No outcome of interest
Trefz 2005 [61]	No quantitative data / insufficient data
Trefz 2009 [62]	No outcome of interest
Trefz 2010 [63]	No outcome of interest
Trefz 2015 [64]	No quantitative data / insufficient data
Ünal 2015 [65]	No outcome of interest
Van Vliet 2015 [66]	No outcome of interest
Vernon 2010 [67]	No outcome of interest
Waisbren 2021 [68]	No dietary liberalization
Wang 2007 [69]	No outcome of interest
White 2013 [70]	No dietary liberalization
Yano 2016 [71]	No outcome of interest

[1] L. Aldámiz-Echevarría *et al.*, "6R-tetrahydrobiopterin treated PKU patients below 4years of age: Physical outcomes, nutrition and genotype," *Mol. Genet. Metab.*, vol. 115, no. 1, pp. 10–16, 2015.

[2] K. Anjema *et al.*, "Tetrahydrobiopterin responsiveness in phenylketonuria: Prediction with the

48-hour loading test and genotype," *Orphanet J. Rare Dis.*, vol. 8, no. 1, 2013.

[3] K. Anjema *et al.*, "The neonatal tetrahydrobiopterin loading test in phenylketonuria: What is the predictive value?," *Orphanet J. Rare Dis.*, vol. 11, no. 1, Jan. 2016.

[4] A. Baldellou Vázquez, M. I. Salazar García-Blanco, M. P. Ruiz-Echarri Zalaya, C. Campos Calleja, L. Ruiz Desviat, and M. Ugarte Pérez, "Tratamiento de la hiperfenilalaninemia por déficit de fenilalanina hidroxilasa con tetrahidrobiopterina. ¿Cuándo y cómo?," *An. Pediatr.*, vol. 64, no. 2, pp. 146–152, 2006.

[5] A. Bélanger-Quintana *et al.*, "Spanish BH4-responsive phenylalanine hydroxylase-deficient patients: Evolution of seven patients on long-term treatment with tetrahydrobiopterin," *Mol. Genet. Metab.*, vol. 86, no. SUPPL., pp. 61–66, 2005.

[6] M. D. Bóveda *et al.*, "The tetrahydrobiopterin loading test in 36 patients with hyperphenylalaninaemia: evaluation of response and subsequent treatment.," *J. Inherit. Metab. Dis.*, vol. 30, no. 5, p. 812, 2007.

[7] T. V. Bushueva *et al.*, "Open, non-comparative phase III clinical study to evaluate the efficacy and safety of sapropterin in patients with phenylketonuria and hyperphenylalaninemia," *Vestn. Ross. Akad. Meditsinskikh Nauk*, no. 7–8, pp. 69–77, 2014.

[8] A. Burlina and N. Blau, "Effect of BH4 supplementation on phenylalanine tolerance," *J. Inherit. Metab. Dis.*, vol. 32, no. 1, pp. 40–45, 2009.

[9] B. K. Burton *et al.*, "The response of patients with phenylketonuria and elevated serum phenylalanine to treatment with oral sapropterin dihydrochloride (6R-tetrahydrobiopterin): A phase II, multicentre, open-label, screening study," *J. Inherit. Metab. Dis.*, vol. 30, no. 5, pp. 700–707, Oct. 2007.

[10] B. K. Burton, H. Bausell, R. Katz, H. LaDuca, and C. Sullivan, "Sapropterin therapy increases stability of blood phenylalanine levels in patients with BH4-responsive phenylketonuria (PKU)," *Mol. Genet. Metab.*, vol. 101, no. 2–3, pp. 110–114, Oct. 2010.

[11] B. K. Burton *et al.*, "Safety of extended treatment with sapropterin dihydrochloride in patients with phenylketonuria: Results of a phase 3b study," *Mol. Genet. Metab.*, vol. 103, no. 4, pp. 315–322, Aug. 2011.

[12] B. Burton *et al.*, "A randomized, placebo-controlled, double-blind study of sapropterin to treat ADHD symptoms and executive function impairment in children and adults with sapropterin-responsive phenylketonuria," *Mol. Genet. Metab.*, vol. 114, no. 3, pp. 415–424, Mar. 2015.

[13] J. Campistol, M. J. González, A. P. Gutiérrez, and M. A. Vilaseca, "Tratamiento y control de los pacientes con fenilcetonuria: resultados del Grupo Colaborativo de Unidades de Seguimiento en España," *Med. Clin. (Barc).*, vol. 138, no. 5, pp. 185–191, Mar. 2012.

[14] C. Cazzorla *et al.*, "Quality of Life (QoL) assessment in a cohort of patients with Phenylketonuria," *BMC Public Health*, vol. 14, 2014.

[15] S. E. Christ, A. J. Moffitt, D. Peck, and D. A. White, "The effects of tetrahydrobiopterin (BH4) treatment on brain function in individuals with phenylketonuria," *NeuroImage Clin.*, vol. 3, pp. 539–547, 2013.

[16] H. E. Clocksin, Z. W. Hawks, D. A. White, and S. E. Christ, "Inter- and intra-tract analysis of white matter abnormalities in individuals with early-treated phenylketonuria (PKU)," *Mol. Genet. Metab.*, vol. 132, no. 1, pp. 11–18, Jan. 2021.

[17] J. L. Cohen-Pfeffer *et al.*, "A randomized, placebo-controlled, double-blind study of

sapropterin to treat symptoms of ADHD and executive dysfunction in children and adolescents with phenylketonuria," *J. Inherit. Metab. Dis.*, vol. 38, no. S1, pp. 35–378, Sep. 2015.

[18] M. L. Couce, M. D. Bóveda, E. Valerio, A. Pérez-Muñuzuri, and J. M. Fraga, "Long-term pharmacological management of phenylketonuria, including patients below the age of 4 years," in *JIMD Reports*, vol. 2, Springer, 2012, pp. 91–96.

[19] S. A. Doggrell, "Is sapropterin treatment suitable for all subjects with phenylketonuria?," *Expert Opin. Pharmacother.*, vol. 9, no. 1, pp. 145–147, Jan. 2008.

[20] T. D. Douglas, H. A. Jinnah, D. Bernhard, and R. H. Singh, "The effects of sapropterin on urinary monoamine metabolites in phenylketonuria," *Mol. Genet. Metab.*, vol. 109, no. 3, pp. 243–250, 2013.

[21] M. Evans, H. Truby, and A. Boneh, "The relationship between dietary intake, growth and body composition in Phenylketonuria," *Mol. Genet. Metab.*, vol. 122, no. 1–2, pp. 36–42, Sep. 2017.

[22] F. Feillet *et al.*, "Use of sapropterin dihydrochloride in maternal phenylketonuria. A European experience of eight cases," *J. Inherit. Metab. Dis.*, vol. 37, no. 5, pp. 753–762, Sep. 2014.

[23] C. Ficicioglu *et al.*, "A pilot study of fluorodeoxyglucose positron emission tomography findings in patients with phenylketonuria before and during sapropterin supplementation," *J. Clin. Neurol.*, vol. 9, no. 3, pp. 151–156, Jul. 2013.

[24] R. Gassió, M. A. Vilaseca, N. Lambruschini, C. Boix, M. E. Fusté, and J. Campistol, "Cognitive functions in patients with phenylketonuria in long-term treatment with tetrahydrobiopterin," *Mol. Genet. Metab.*, vol. 99, no. SUPPL., 2010.

[25] D. K. Grange *et al.*, "Sapropterin dihydrochloride use in pregnant women with phenylketonuria: An interim report of the PKU MOMS sub-registry," *Mol. Genet. Metab.*, vol. 112, no. 1, pp. 9–16, 2014.

[26] J. B. Hennermann, C. Bührer, N. Blau, B. Vetter, and E. Mönch, "Long-term treatment with tetrahydrobiopterin increases phenylalanine tolerance in children with severe phenotype of phenylketonuria," *Mol. Genet. Metab.*, vol. 86, no. SUPPL., pp. 86–90, 2005.

[27] J. B. Hennermann, S. Roloff, C. Gebauer, B. Vetter, A. von Arnim-Baas, and E. Mönch, "Long-term treatment with tetrahydrobiopterin in phenylketonuria: Treatment strategies and prediction of long-term responders," *Mol. Genet. Metab.*, vol. 107, no. 3, pp. 294–301, Nov. 2012.

[28] S. C. J. Huijbregts *et al.*, "The impact of metabolic control and tetrahydrobiopterin treatment on health related quality of life of patients with early-treated phenylketonuria: A PKU-COBESO study," *Mol. Genet. Metab.*, vol. 125, no. 1–2, pp. 96–103, Sep. 2018.

[29] M. Humphrey, J. Nation, I. Francis, and A. Boneh, "Effect of tetrahydrobiopterin on Phe/Tyr ratios and variation in Phe levels in tetrahydrobiopterin responsive PKU patients," *Mol. Genet. Metab.*, vol. 104, no. 1–2, pp. 89–92, Sep. 2011.

[30] M. Kagnici *et al.*, "The comparison of the effect of tetrahydrobiopterin and phenylalanine deficient diet on oxidative stress in hyperphenylalaninemia," in *Journal of inherited metabolic disease*, 2014, vol. 37, pp. 27–185.

[31] S. Keil *et al.*, "Long-term follow-up and outcome of phenylketonuria patients on sapropterin: A retrospective study," *Pediatrics*, vol. 131, no. 6, Jun. 2013.

[32] D. Kör, B. Ş. Yilmaz, F. D. Bulut, S. Ceylaner, and N. Ö. Mungan, "Improved metabolic control in tetrahydrobiopterin (BH4), responsive phenylketonuria with sapropterin

administered in two divided doses vs. a single daily dose," *J. Pediatr. Endocrinol. Metab.*, vol. 30, no. 7, pp. 713–718, Jul. 2017.

[33] P. Lee *et al.*, "Safety and efficacy of 22 weeks of treatment with sapropterin dihydrochloride in patients with phenylketonuria," *Am. J. Med. Genet. Part A*, vol. 146, no. 22, pp. 2851–2859, Nov. 2008.

[34] O. Leuret *et al.*, "Efficacy and safety of BH4 before the age of 4 years in patients with mild phenylketonuria," *J. Inherit. Metab. Dis.*, vol. 35, no. 6, pp. 975–981, Nov. 2012.

[35] H. L. Levy *et al.*, "Efficacy of sapropterin dihydrochloride (tetrahydrobiopterin, 6R-BH4) for reduction of phenylalanine concentration in patients with phenylketonuria: a phase III randomised placebo-controlled study," *Lancet*, vol. 370, no. 9586, pp. 504–510, Aug. 2007.

[36] M. Lou Lindegren, S. Krishnaswami, T. Reimschisel, C. Fonnesbeck, N. A. Sathe, and M. L. McPheeters, "A systematic review of BH4 (Sapropterin) for the adjuvant treatment of phenylketonuria," in *JIMD Reports*, vol. 8, Springer, 2013, pp. 109–119.

[37] N. Longo *et al.*, "Long-term developmental progression in infants and young children taking sapropterin for phenylketonuria: A two-year analysis of safety and efficacy," *Genet. Med.*, vol. 17, no. 5, pp. 365–373, May 2015.

[38] N. Longo *et al.*, "Long-term safety and efficacy of sapropterin: The PKUDOS registry experience," *Mol. Genet. Metab.*, vol. 114, no. 4, pp. 557–563, Apr. 2015.

[39] K. D. Moseley, M. J. Ottina, C. G. Azen, and S. Yano, "Pilot study to evaluate the effects of tetrahydrobiopterin on adult individuals with phenylketonuria with measurable maladaptive behaviors," *CNS Spectr.*, vol. 20, no. 2, pp. 157–163, Feb. 2015.

[40] A. C. Muntau *et al.*, "Tetrahydrobiopterin as an Alternative Treatment for Mild Phenylketonuria," *N. Engl. J. Med.*, vol. 347, no. 26, pp. 2122–2132, Dec. 2002.

[41] A. C. Muntau, D. Rogoff, and J. Zschocke, "Efficacy and safety of sapropterin dihydrochloride in patients with phenylketonuria aged less than 4 years old: Results from the SPARK study, a phase IIIb, multicentre, open-label, randomized, controlled study," in *Journal of inherited metabolic disease*, 2014, vol. 37, pp. 27–185.

[42] A. C. Muntau *et al.*, "The sixth interim analysis of the Kuvan® Adult Maternal Paediatric European Registry (KAMPER): Interim results in PKU and BH4 deficiency patients," *J. Inherit. Metab. Dis.*, vol. 39, no. S1, pp. 35–284, Sep. 2016.

[43] A. C. Muntau *et al.*, "Efficacy, safety and population pharmacokinetics of sapropterin in PKU patients <4 years: results from the SPARK open-label, multicentre, randomized phase IIIb trial," *Orphanet J. Rare Dis.*, vol. 12, no. 1, Mar. 2017.

[44] A. Ormazabal *et al.*, "Platelet serotonin concentrations in PKU patients under dietary control and tetrahydrobiopterin treatment," *J. Inherit. Metab. Dis.*, vol. 28, no. 6, pp. 863–870, Dec. 2005.

[45] S. Prasad *et al.*, "Neuropsychiatric outcomes in PKU patients treated with sapropterin: Results from the randomized, controlled PKU ascend (PKU 016) trial," in *Journal of Inherited Metabolic Disease*, 2013, vol. 36, no. S2, pp. 91–342.

[46] J. Qu *et al.*, "Efficacy and safety of sapropterin dihydrochloride in patients with phenylketonuria: A meta-analysis of randomized controlled trials," *British Journal of Clinical Pharmacology*, vol. 85, no. 5. Blackwell Publishing Ltd, pp. 893–899, 01-May-2019.

[47] F. Rutsch *et al.*, "Efficacy & safety of sapropterin in PKU patients," in *Journal of Inherited*

Metabolic Disease, 2018, vol. 41, no. S1, pp. 37–219.

[48] H. Shintaku *et al.*, "Long-Term Treatment and Diagnosis of Tetrahydrobiopterin-Responsive Hyperphenylalaninemia with A Mutant Phenylalanine Hydroxylase Gene," *Pediatr. Res.*, vol. 55, no. 3, pp. 425–430, Mar. 2004.

[49] H. Shintaku, H. Fujioka, Y. Sawada, M. Asada, and T. Yamano, "Plasma biopterin levels and tetrahydrobiopterin responsiveness," *Mol. Genet. Metab.*, vol. 86, no. SUPPL., pp. 104–106, 2005.

[50] H. Shintaku and T. Ohura, "Sapropterin is safe and effective in patients less than 4-years-old with bh4-responsive phenylalanine hydroxylase deficiency," *J. Pediatr.*, vol. 165, no. 6, pp. 1241–1244, Dec. 2014.

[51] R. H. Singh and M. E. Quirk, "Using change in plasma phenylalanine concentrations and ability to liberalize diet to classify responsiveness to tetrahydrobiopterin therapy in patients with phenylketonuria," *Mol. Genet. Metab.*, vol. 104, no. 4, pp. 485–491, Dec. 2011.

[52] U. R. Somaraju and M. Merrin, "Sapropterin dihydrochloride for phenylketonuria," *Cochrane Database of Systematic Reviews*, no. 4. 2009.

[53] U. R. Somaraju and M. Merrin, "Sapropterin dihydrochloride for phenylketonuria," in *Cochrane Database of Systematic Reviews*, no. 6, John Wiley & Sons, Ltd, 2010.

[54] U. R. Somaraju and M. Merrin, "Sapropterin dihydrochloride for phenylketonuria," *Cochrane Database of Systematic Reviews*, vol. 2012, no. 12. John Wiley and Sons Ltd, 12-Dec-2012.

[55] U. R. Somaraju and M. Merrin, "Sapropterin dihydrochloride for phenylketonuria," *Cochrane Database of Systematic Reviews*, vol. 2015, no. 3. John Wiley and Sons Ltd, 27-Mar-2015.

[56] C. Sousa Barbosa *et al.*, "Metabolic Control in Patients With Phenylketonuria Pre- and Post-Sapropterin Loading Test," *J. Inborn Errors Metab. Screen.*, vol. 6, p. 232640981878889, Jan. 2018.

[57] S. Stockler-Ipsiroglu *et al.*, "Individualized long-term outcomes in blood phenylalanine concentrations and dietary phenylalanine tolerance in 11 patients with primary phenylalanine hydroxylase (PAH) deficiency treated with Sapropterin-dihydrochloride," *Mol. Genet. Metab.*, vol. 114, no. 3, pp. 409–414, Mar. 2015.

[58] M. Tamura, S. Seki, Y. Kakurai, S. Chikada, and K. Wada, "Sapropterin for phenylketonuria: A Japanese post-marketing surveillance study," *Pediatr. Int.*, vol. 64, no. 1, p. e14939, Jan. 2022.

[59] A. G. Thiele *et al.*, "Nutritional changes and micronutrient supply in patients with phenylketonuria under therapy with tetrahydrobiopterin (BH4)," in *JIMD Reports*, vol. 9, Springer, 2013, pp. 31–40.

[60] A. G. Thiele *et al.*, "The challenge of long-term tetrahydrobiopterin (BH4) therapy in phenylketonuria: Effects on metabolic control, nutritional habits and nutrient supply," *Mol. Genet. Metab. Reports*, vol. 4, pp. 62–67, Jul. 2015.

[61] F. K. Trefz, D. Scheible, G. Frauendienst-Egger, H. Korall, and N. Blau, "Long-term treatment of patients with mild and classical phenylketonuria by tetrahydrobiopterin," *Mol. Genet. Metab.*, vol. 86, no. SUPPL., pp. 75–80, 2005.

[62] F. K. Trefz *et al.*, "Efficacy of Sapropterin Dihydrochloride in Increasing Phenylalanine Tolerance in Children with Phenylketonuria: A Phase III, Randomized, Double-Blind, Placebo-Controlled Study," *J. Pediatr.*, vol. 154, no. 5, 2009.

[63] F. K. Trefz, D. Scheible, and G. Frauendienst-Egger, “Long-term follow-up of patients with phenylketonuria receiving tetrahydrobiopterin treatment,” *J. Inherit. Metab. Dis.*, vol. 33, no. SUPPL. 3, 2010.

[64] F. K. Trefz *et al.*, “The Kuvan® adult maternal paediatric european registry (KAMPER) multinational observational study: Baseline and 1-year data in phenylketonuria patients responsive to sapropterin,” in *JIMD Reports*, vol. 23, Springer, 2015, pp. 35–43.

[65] Ö. Ünal *et al.*, “Sapropterin dihydrochloride treatment in Turkish hyperphenylalaninemic patients under age four,” 2015.

[66] D. van Vliet *et al.*, “BH4 treatment in BH4-responsive PKU patients: Preliminary data on blood prolactin concentrations suggest increased cerebral dopamine concentrations,” *Mol. Genet. Metab.*, vol. 114, no. 1, pp. 29–33, Jan. 2015.

[67] H. J. Vernon, C. B. Koerner, M. R. Johnson, A. Bergner, and A. Hamosh, “Introduction of sapropterin dihydrochloride as standard of care in patients with phenylketonuria,” *Mol. Genet. Metab.*, vol. 100, no. 3, pp. 229–233, Jul. 2010.

[68] S. Waisbren *et al.*, “Long-term preservation of intellectual functioning in sapropterin-treated infants and young children with phenylketonuria: A seven-year analysis,” *Mol. Genet. Metab.*, vol. 132, no. 2, pp. 119–127, Feb. 2021.

[69] L. Wang *et al.*, “Mutations in the regulatory domain of phenylalanine hydroxylase and response to tetrahydrobiopterin,” *Genet. Test.*, vol. 11, no. 2, pp. 174–178, Jun. 2007.

[70] D. A. White *et al.*, “White matter integrity and executive abilities following treatment with tetrahydrobiopterin (BH4) in individuals with phenylketonuria,” *Mol. Genet. Metab.*, vol. 110, no. 3, pp. 213–217, Nov. 2013.

[71] S. Yano, K. Moseley, X. Fu, and C. Azen, “Evaluation of tetrahydrobiopterin therapy with large neutral amino acid supplementation in phenylketonuria: Effects on potential peripheral biomarkers, melatonin and dopamine, for brain monoamine neurotransmitters,” *PLoS One*, vol. 11, no. 8, Aug. 2016.
