# Supplementary material for: Dietary Liberalization in Tetrahydrobiopterin-Treated PKU Patients: Does It Improve Outcomes?
Source: Nutrients. 2022 Sep 19;14(18):3874. doi: 10.3390/nu14183874 (PMC9504284; doi:10.3390/nu14183874)
Supplement: Supplementary file 1 [file nutrients-14-03874-s001.zip › Supplementary file S6 - Risk of bias in individual studies.pdf]

Supplementary file S6 - Risk of bias in individual studies

	Study participation	Study attrition	Prognostic factor measurement	Outcome measurement	Study confounding	Statistical analysis and reporting
Lambruschini 2005	orange	green	green	orange	orange	green
Muntau 2021	orange	orange	green	orange	orange	orange

Table S6.1. Risk of bias in individual studies for neurocognitive outcome, assessed by the Quality In Prognosis Studies (QUIPS) tool. Each possible area of bias was scored as low risk of bias (green), moderate risk of bias (orange) of high risk of bias (red).

	Study participation	Study attrition	Prognostic factor measurement	Outcome measurement	Study confounding	Statistical analysis and reporting
Lambruschini 2005	orange	green	green	orange	orange	green
Singh 2010	orange	green	green	green	orange	green
Aldámiz-Echevarría 2013	orange	green	orange	orange	orange	green
Tansek 2016	orange	green	green	orange	orange	orange
Scala 2015	orange	green	green	red	orange	green
Evers 2018	green	green	orange	orange	orange	green
Muntau 2021	orange	orange	green	green	orange	orange

Table S6.2. Risk of bias in individual studies for anthropometric measurements, assessed by the Quality In Prognosis Studies (QUIPS) tool. Each possible area of bias was scored as low risk of bias (green), moderate risk of bias (orange) of high risk of bias (red).

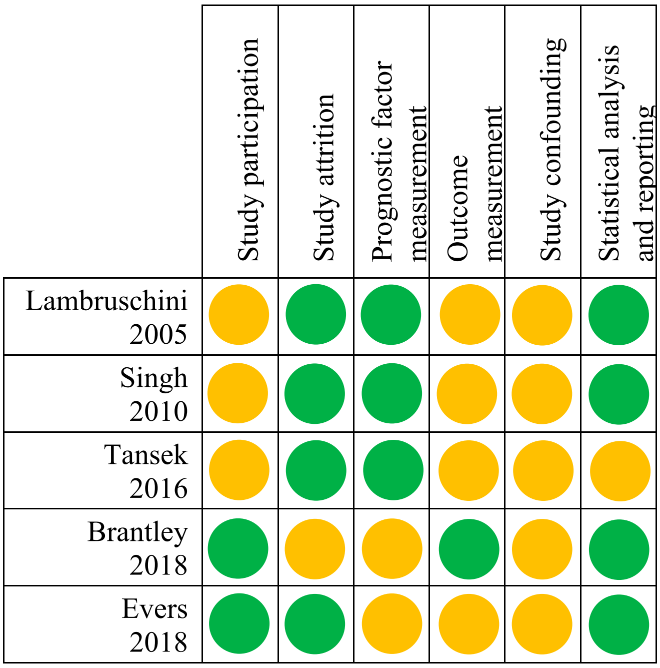

Table S6.3. Risk of bias in individual studies for nutritional biomarkers, assessed by the Quality In Prognosis Studies (QUIPS) tool. Each possible area of bias was scored as low risk of bias (green), moderate risk of bias (orange) of high risk of bias (red).

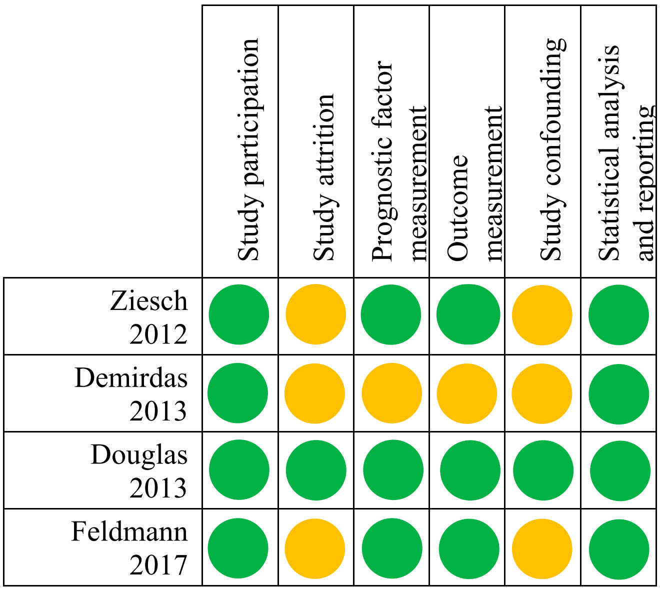

Table S6.4. Risk of bias in individual studies for quality of life, assessed by the Quality In Prognosis Studies (QUIPS) tool. Each possible area of bias was scored as low risk of bias (green), moderate risk of bias (orange) of high risk of bias (red).
