# Supplementary material for: Dietary Liberalization in Tetrahydrobiopterin-Treated PKU Patients: Does It Improve Outcomes?
Source: Nutrients. 2022 Sep 19;14(18):3874. doi: 10.3390/nu14183874 (PMC9504284; doi:10.3390/nu14183874)
Supplement: Supplementary file 1 [file nutrients-14-03874-s001.zip › Supplementary file S7 - Funnel plots.pdf]

Supplementary file S7 - Funnel plots of primary meta-analyses

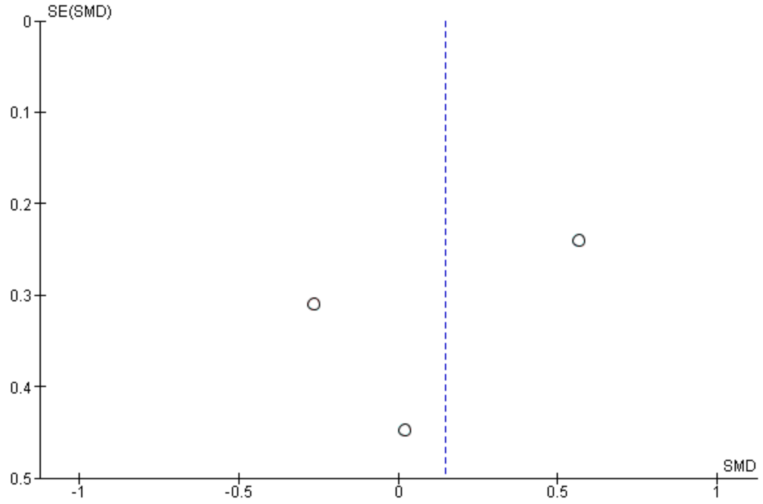

Figure S7.1. Funnel plot of the primary within-subject meta-analysis for weight.

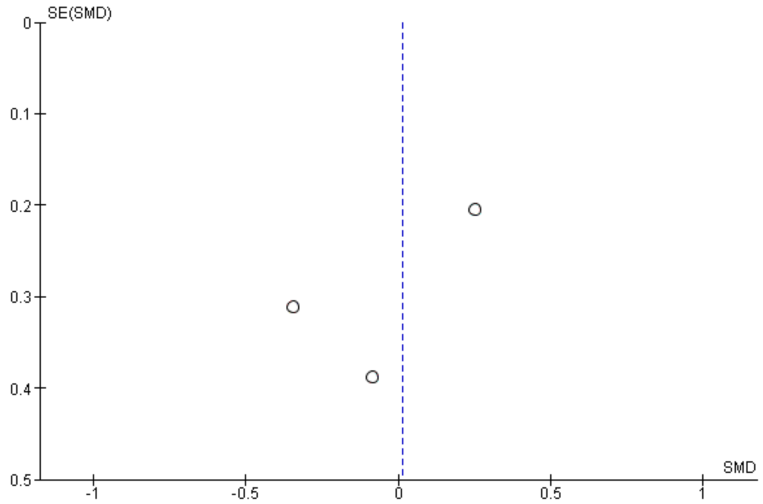

Figure S7.2. Funnel plot of the primary between-subject meta-analysis for weight.

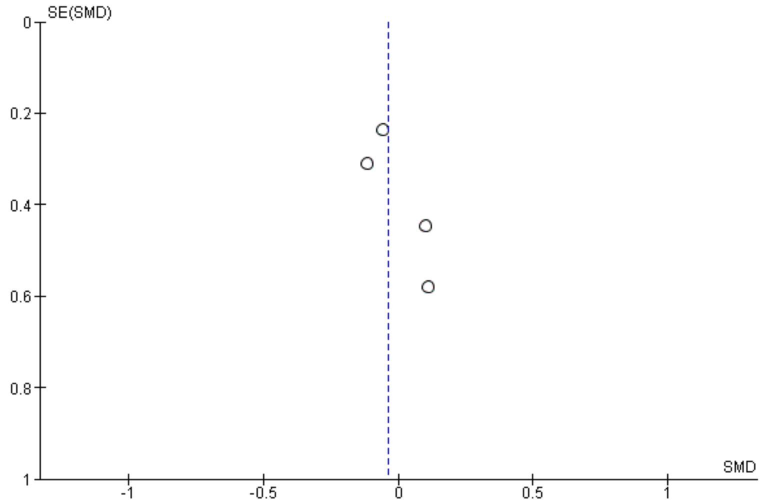

Figure S7.3. Funnel plot of the primary within-subject meta-analysis for BMI.

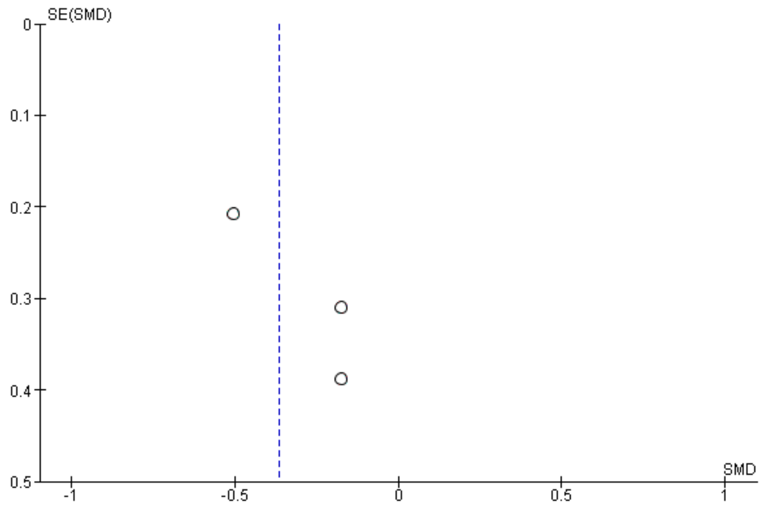

Figure S7.4. Funnel plot of the primary between-subject meta-analysis for BMI.

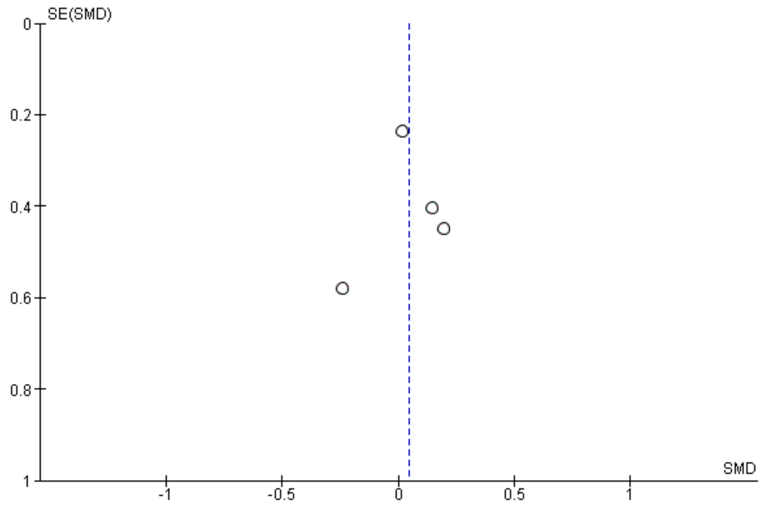

Figure S7.5. Funnel plot of the primary within-subject meta-analysis for height.

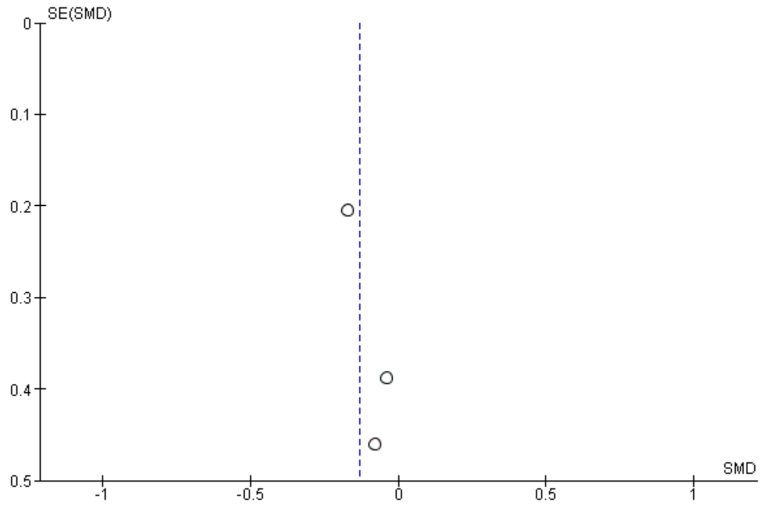

Figure S7.6. Funnel plot of the primary between-subject meta-analysis for height.

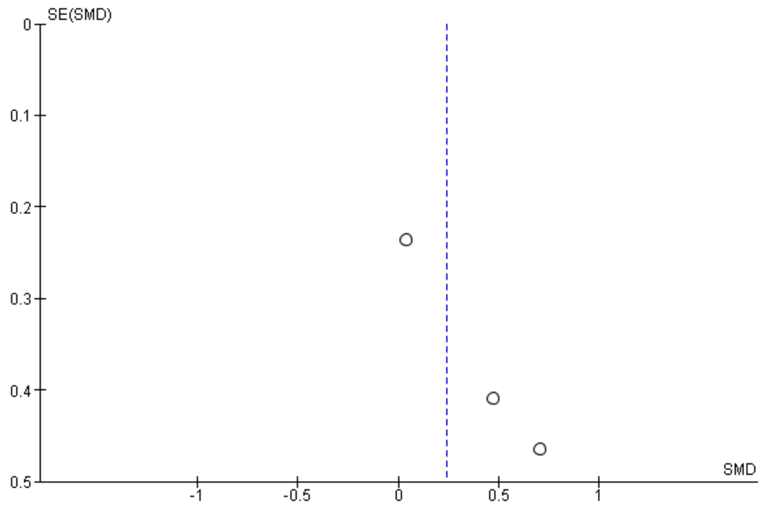

Figure S7.7. Funnel plot of the primary within-subject meta-analysis for growth velocity.

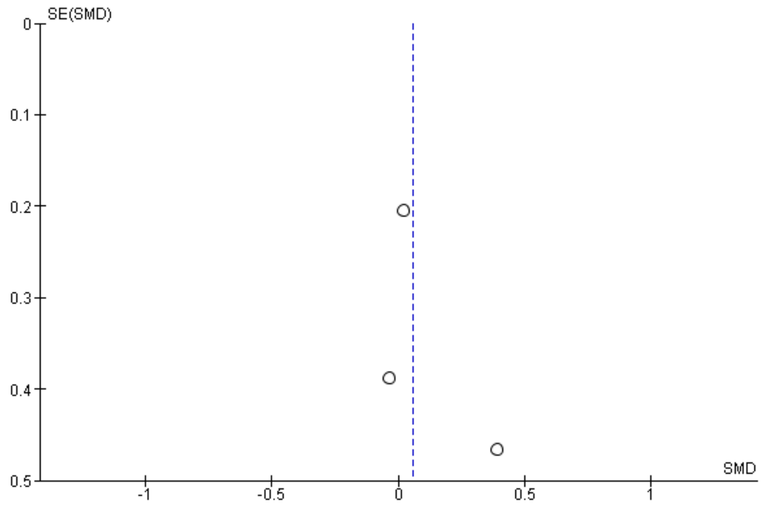

Figure S7.8. Funnel plot of the primary between-subject meta-analysis for growth velocity.

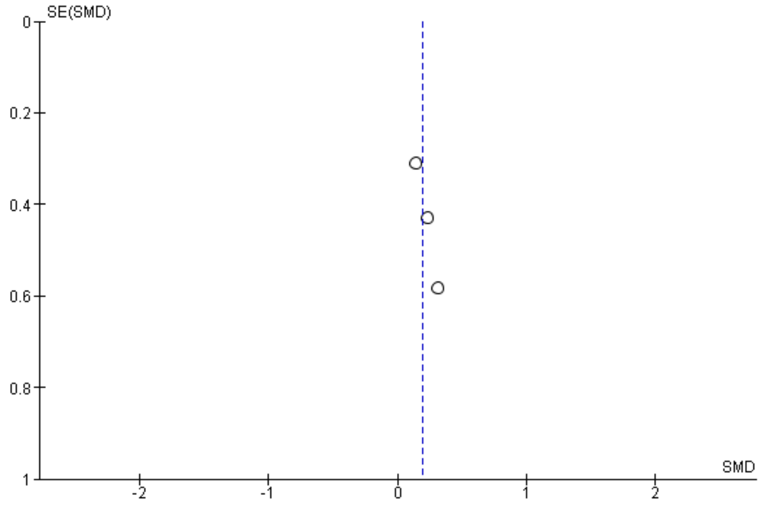

Figure S7.9. Funnel plot of the primary within-subject meta-analysis for blood albumin concentrations.

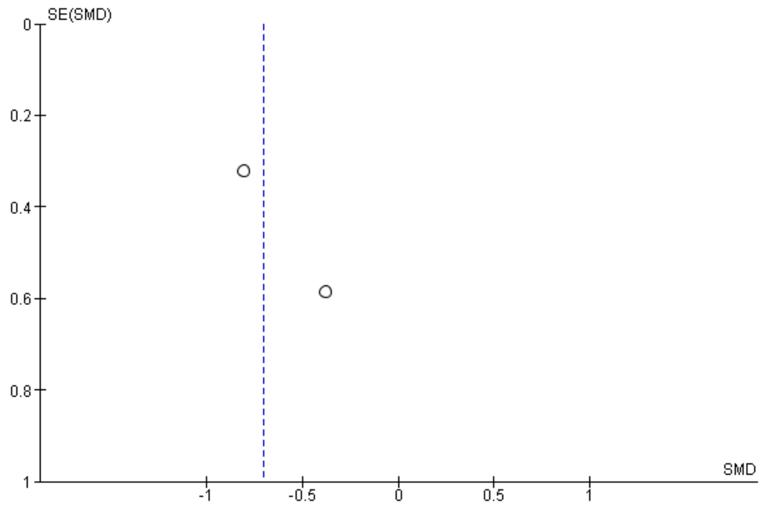

Figure S7.10. Funnel plot of the primary within-subject meta-analysis for blood cholesterol concentrations.

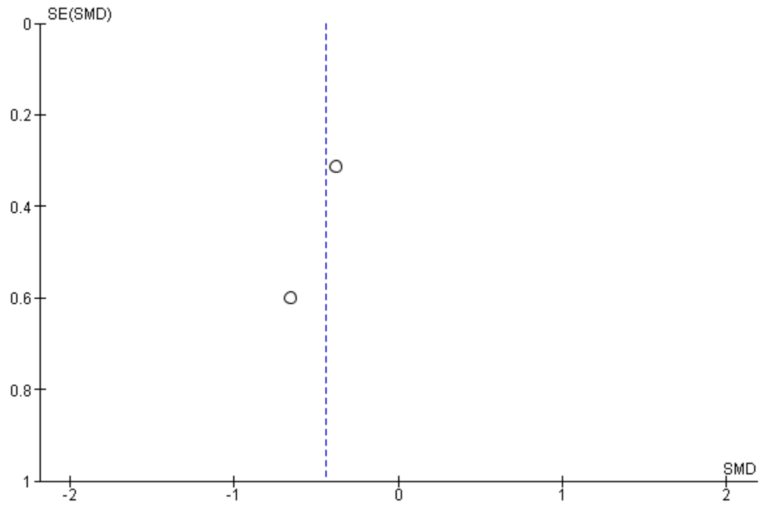

Figure S7.11. Funnel plot of the primary within-subject meta-analysis for blood haemoglobin concentrations.

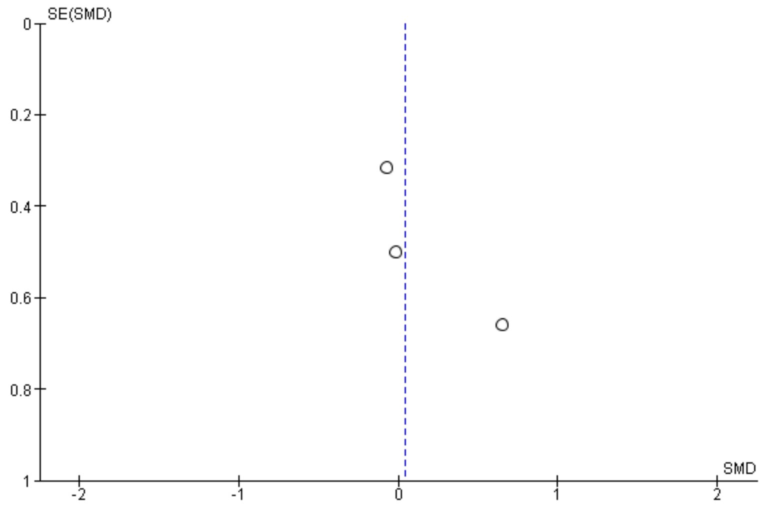

Figure S7.12. Funnel plot of the primary within-subject meta-analysis for generic health-related quality of life – patient report.

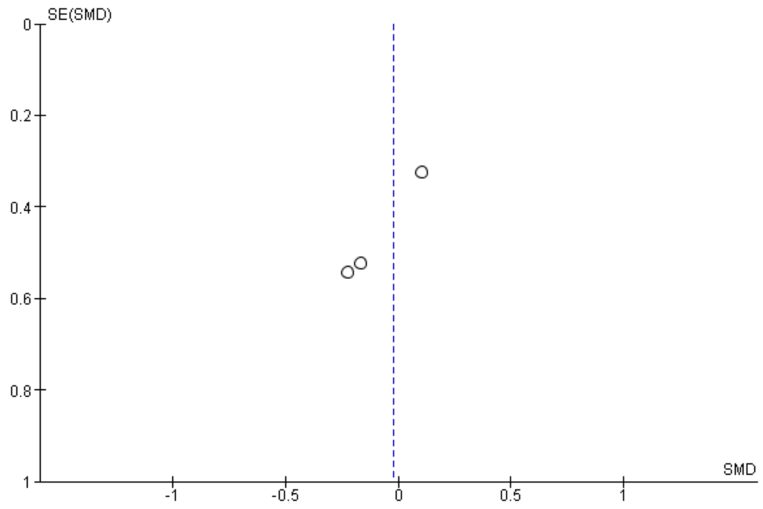

Figure S7.13. Funnel plot of the primary between-subject meta-analysis for generic health-related quality of life – patient report.

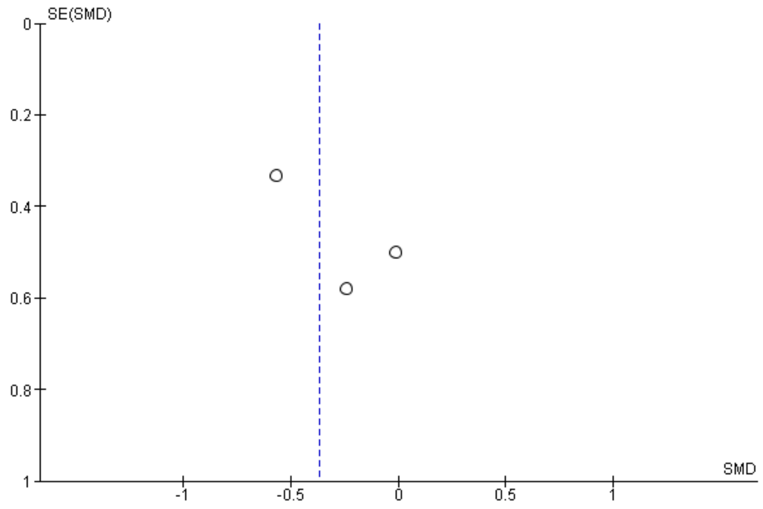

Figure S7.14. Funnel plot of the primary within-subject meta-analysis for generic health-related quality of life – proxy report.

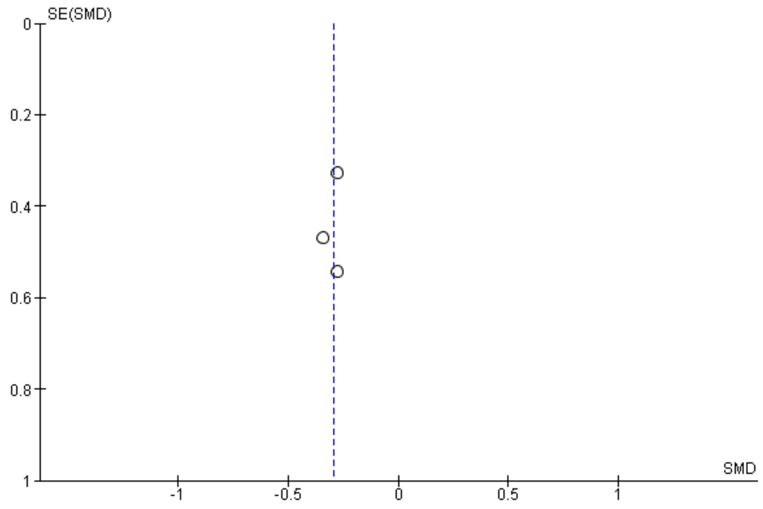

Figure S7.15. Funnel plot of the primary between-subject meta-analysis for generic health-related quality of life – proxy report.
