# Supplementary material for: Dietary Liberalization in Tetrahydrobiopterin-Treated PKU Patients: Does It Improve Outcomes?
Source: Nutrients. 2022 Sep 19;14(18):3874. doi: 10.3390/nu14183874 (PMC9504284; doi:10.3390/nu14183874)
Supplement: Supplementary file 1 [file nutrients-14-03874-s001.zip › Supplementary file S8 - GRADE ratings.pdf]

Supplementary file S8 – GRADE ratings

Outcome	GRADE rating<br>High / moderate / low / very low	Explanation<br>Reasons for lowering or raising certainty
**IQ**	Very low	Imprecision due to low sample size (n < 50)
**Development**	Low	
**Weight – within-subject**	Low	
**Weight – between-subject**	Low	
**Body mass index – within-subject**	Low	
**Body mass index – between-subject**	Low	
**Height – within-subject**	Low	
**Height – between-subject**	Low	
**Growth velocity – within-subject**	Low	
**Growth velocity – between-subject**	Low	
**Head circumference – within-subject**	Low	
**Brachial muscular area – within-subject**	Very low	Imprecision due to low sample size (n < 50)
**Brachial adipose area – within-subject**	Very low	Imprecision due to low sample size (n < 50)
**Cholesterol – within-subject**	Very low	Imprecision due to low sample size (n < 50)
**Cholesterol – between-subject**	Very low	Imprecision due to low sample size (n < 50)
**Triglycerides – within-subject**	Very low	Imprecision due to low sample size (n < 50)
**Triglycerides – between-subject**	Very low	Imprecision due to low sample size (n < 50)
**Iron – within-subject**	Very low	Imprecision due to low sample size (n < 50)
**Iron – between-subject**	Very low	Imprecision due to low sample size (n < 50)
**Transferrin – within-subject**	Very low	Imprecision due to low sample size (n < 50)
**Transferrin – between-subject**	Very low	Imprecision due to low sample size (n < 50)
**Ferritin – within-subject**	Very low	Imprecision due to low sample size (n < 50)
**Albumin – within-subject**	Very low	Imprecision due to low sample size (n < 50)
**Albumin – between-subject**	Very low	Imprecision due to low sample size (n < 50)
**Total protein – within-subject**	Very low	Imprecision due to low sample size (n < 50)
**Tranthyretin – within-subject**	Very low	Imprecision due to low sample size (n < 50)
**Vitamin A – within-subject**	Very low	Imprecision due to low sample size (n < 50)
**Vitamin B6 – within-subject**	Very low	Imprecision due to low sample size (n < 50)
**Vitamin B6 – between-subject**	Very low	Imprecision due to low sample size (n < 50)
**Folate – within-subject**	Very low	Imprecision due to low sample size (n < 50)

**Folate – between-subject**	Very low	Imprecision due to low sample size (n < 50)
**Vitamin B12 – within-subject**	Very low	Imprecision due to low sample size (n < 50)
**Vitamin B12 – between-subject**	Very low	Imprecision due to low sample size (n < 50)
**MMA – within-subject**	Very low	Imprecision due to low sample size (n < 50)
**MMA – between-subject**	Very low	Imprecision due to low sample size (n < 50)
**Calcifedol – within-subject**	Very low	Imprecision due to low sample size (n < 50)
**Calcifedol – between-subject**	Very low	Imprecision due to low sample size (n < 50)
**Vitamin E – within-subject**	Very low	Imprecision due to low sample size (n < 50)
**Calcium – within-subject**	Very low	Imprecision due to low sample size (n < 50)
**Calcium – between-subject**	Very low	Imprecision due to low sample size (n < 50)
**Phosphate – within-subject**	Very low	Imprecision due to low sample size (n < 50)
**Phosphate – between-subject**	Very low	Imprecision due to low sample size (n < 50)
**Selenium – within-subject**	Very low	Imprecision due to low sample size (n < 50)
**Zinc – within-subject**	Very low	Imprecision due to low sample size (n < 50)
**Hemoglobin – within-subject**	Very low	Imprecision due to low sample size (n < 50)
**Hemoglobin – between-subject**	Very low	Imprecision due to low sample size (n < 50)
**Haematocrit – within-subject**	Very low	Imprecision due to low sample size (n < 50)
**Generic HR-QoL – patient report – within-subject**	Very low	Imprecision due to low sample size (n < 50)
**Generic HR-QoL – patient report – between-subject**	Very low	Imprecision due to low sample size (n < 50)
**Generic HR-QoL – proxy report – within-subject**	Very low	Imprecision due to low sample size (n < 50)
**Generic HR-QoL – proxy report – between-subject**	Very low	Imprecision due to low sample size (n < 50)
**Specified HR-QoL – patient report – within-subject**	Very low	Imprecision due to low sample size (n < 50)
**Specified HR-QoL – patient report – between-subject**	Very low	Imprecision due to low sample size (n < 50)
**Specified HR-QoL – proxy report – within-subject**	Very low	Imprecision due to low sample size (n < 50)
**Specified HR-QoL – proxy report – between-subject**	Very low	Imprecision due to low sample size (n < 50)
**Parental QoL – between-subject**	Very low	Imprecision due to low sample size (n < 50)

Table S8.1. Overview of the GRADE rating of each parameter. The initial rating was "low" for all parameters due to mostly observational study designs. This rating was then adjusted for reasons outlined in the last column.
