# Supplementary material for: Dietary Liberalization in Tetrahydrobiopterin-Treated PKU Patients: Does It Improve Outcomes?
Source: Nutrients. 2022 Sep 19;14(18):3874. doi: 10.3390/nu14183874 (PMC9504284; doi:10.3390/nu14183874)
Supplement: Supplementary file 1 [file nutrients-14-03874-s001.zip › Supplementary file S9 - Studies with lower Phe levels.pdf]

Supplementary file S9 – Studies with lower phenylalanine concentrations after BH4 treatment

Study	Main findings	Cohort characteristics: Number of patients	Age at baseline (years)	Follow-up time (months)	Blood Phe concentrations: Baseline (µmol/l)	Follow-up (µmol/l)	Change
	**Neuro-imaging**						
Christ 2013	Activation changed significantly in one out of 15 regions of interest.	12	23.6 ± 8.8	6	681	582	-15%
White 2013	Mean diffusivity improved in two out of 10 regions of interest.	12	18.2 ± 9.6	6	653	409	-37%
Clocksin 2021	White matter integrity improved significantly in 11 out 14 tracts.	8	22.5 ± 10.1	6.4[a]	693	375	-46%
	**IQ**						
White 2013	IQ did not significantly change after starting BH_4 treatment, and was not significantly different compared to controls.	12	18.2 ± 9.6	6	653	409	-37%
	**Executive functioning**						
Christ 2013	Working memory significantly improved after starting BH4 treatment. Outcome was worse than controls at baseline, but there was no significant difference anymore after staring BH4 treatment.	12	23.6 ± 8.8	6	681	582	-18%
White 2013	Executive abilities did not significantly change after starting BH4 treatment, and were not significantly different compared to controls.	12	18.2 ± 9.6	6	653	409	-37%
Burton 2015	- In patients < 18 years, global executive functioning as well as one of the two subscales (metacognition index) was significantly better compared to controls.<br>- In patients > 18 years, global executive functioning and neither of the two subscales were significantly different compared to controls<br>- Improvement of global functioning was not significantly different compared to controls.	61 / 57[b]	19.6 ± 10.1 / 20.2 ± 10.1[b]	6 / 3[b]	680 / 703	470 / 484	-31% / -31%
	**Mental health**						
Burton 2015	- Total ADHD score did not significantly differ compared to controls. However, one of the two subscales (inattention) was significantly better compared to controls.<br>- In patients < 18 years, total ADHD score and one of the two subscales (inattention) were significantly better compared to controls.<br>- In patients > 18 years, total ADHD score and neither of the two subscales were significantly better compared to controls.<br>- Total ADHD score and the inattention subscale significantly decreased after starting BH4 treatment relative to the placebo group.	61 / 57[b]	19.6 ± 10.1 / 20.2 ± 10.1[b]	6 / 3[b]	680 / 703	470 / 484	-31% / -31%

Table S9.1. Summary of outcomes in studies that investigated the effects of lower blood Phe concentrations following BH_4 treatment. Change in dietary Phe was not reported in these studies. [a]Mean follow-up time (different follow-up times for different patients). [b]Results for two separate cohorts (BH_4 treatment for 26 weeks / BH_4 treatment for 13 weeks).
